# PCV2 Induced Endothelial Derived IL-8 Affects MoDCs Maturation Mainly via NF-κB Signaling Pathway

**DOI:** 10.3390/v16040646

**Published:** 2024-04-22

**Authors:** Mengyu Zhang, Weicheng Xu, Ning Yang, Zhuowei Li, Shuanghai Zhou, Xuewei Liu, Jianfang Wang, Huanrong Li

**Affiliations:** College of Animal Science and Technology, Beijing University of Agriculture, No. 7 Beinong Road, Beijing 102206, China; zhangmengyu316@163.com (M.Z.); 17667421064@163.com (W.X.); yangning01@caas.cn (N.Y.); lizhuowei_vet@163.com (Z.L.); shhaizhou@bua.edu.cn (S.Z.); liuxuewei66@163.com (X.L.)

**Keywords:** porcine circovirus type 2, monocyte-derived dendritic cells, endothelial derived IL-8, NF-κB signal pathway, JAK2-STAT3 pathway

## Abstract

Porcine circovirus type 2 (PCV2) infection can cause immunosuppressive diseases in pigs. Vascular endothelial cells (VECs), as the target cells for PCV2, play an important role in the immune response and inflammatory regulation. Endothelial IL-8, which is produced by porcine hip artery endothelial cells (PIECs) infected with PCV2, can inhibit the maturation of monocyte-derived dendritic cells (MoDCs). Here, we established a co-culture system of MoDCs and different groups of PIECs to further investigate the PCV2-induced endothelial IL-8 signaling pathway that drives the inhibition of MoDC maturation. The differentially expressed genes related to MoDC maturation were mainly enriched in the NF-κB and JAK2-STAT3 signaling pathways. Both the NF-κB related factor RELA and JAK2-STAT3 signaling pathway related factors (IL2RA, JAK, STAT2, STAT5, IL23A, IL7, etc.) decreased significantly in the IL-8 up-regulated group, and increased significantly in the down-regulated group. The expression of NF-κB p65 in the IL-8 up-regulated group was reduced significantly, and the expression of IκBα was increased significantly. Nuclear translocation of NF-κB p65 was inhibited, while the nuclear translocation of p-STAT3 was increased in MoDCs in the PCV2-induced endothelial IL-8 group. The results of treatment with NF-κB signaling pathway inhibitors showed that the maturation of MoDCs was inhibited and the expression of IL-12 and GM-CSF at mRNA level were lower. Inhibition of the JAK2-STAT3 signaling pathway had no significant effect on maturation, and the expression of IL-12 and GM-CSF at mRNA level produced no significant change. In summary, the NF-κB signaling pathway is the main signaling pathway of MoDC maturation, and is inhibited by the PCV2-induced up-regulation of endothelial-derived IL-8.

## 1. Introduction

Porcine circovirus type 2 (PCV2) belongs to the family of circoviruses and is a small, non-enveloped, single-stranded (ss) DNA virus [1]. It is a major factor in porcine circovirus disease (PCVD) outbreaks, which poses a significant economic and health threat in swine production worldwide [2]. PCVDs include weight loss or postweaning multisystemic wasting syndrome (PMWS), respiratory disease, enteritis, reproductive failure, porcine dermatitis nephropathy syndrome (PDNS), myocarditis/vasculitis, and exudative dermatitis [3].

PCV2 can exist in the cardiomyocytes, hepatocytes, macrophages, and vascular endothelial cells (VECs) of infected pigs [4]. PCV2 infection leads to vascular lesions and the degeneration of endothelial cells [5], and PCV2-infected endothelial cells can also modulate leukocyte migration, cause inflammatory responses, and induce endothelial cell-derived IL-8 production [6]. The function of endothelial IL-8 is different from that of monocyte derived IL-8 due to their structural differences, as the former has an extra five amino acid extension of AVLPR at the N-terminus [7].

DCs are the most efficient antigen-presenting cells (APCs) linking the innate and adaptive immune systems. They possess a unique ability to effectively trigger the initial T-lymphocyte-stimulated memory response [8]. When immature DCs are stimulated by an antigen, they first adhere to endothelial cells and migrate to peripheral lymphoid tissues to mount an immune response [9]. The differentiation and function of DCs are easily influenced by cellular and microenvironmental changes. For example, the presence of a tumor environment inhibits DC maturation in colorectal cancer, whereas the splenic mesenchymal microenvironment can modulate the function of virus-activated plasmacytoid dendritic cells (pDCs) [10,11]. In the context of PCV2 infection, IL-8 secreted by porcine hip artery endothelial cells (PIECs) is up-regulated, leading to inhibition of DC maturation, and subsequent immune suppression [12]. Maturation is central to the functional development of DCs. Immature DCs have strong phagocytosis, whereas mature DCs have reduced phagocytosis and increased antigen-presenting capacity. Interestingly, the signaling pathways involved may vary due to factors affecting DC maturation. LPS induces DC maturation through activation of p38 SAPK, the classical MAP kinase pathway (MEK/ERK), and the NF-κB pathway [13]. MicroRNA let-7i can regulate the maturation of DCs by targeting interleukin-10 through the Janus kinase 1 (JAK1) signal transducer and transcriptional activator 3 (STAT3) signaling pathways [14]. The differentiation and maturation process of mouse DCs is broadcast through the TLR-4/NF-κB pathway, and CD40 ligand and TNF-α can regulate DC function and maturation through activating NF-κB [15,16]. However, the signaling mechanism underlying the inhibition of DC maturation by PCV2-induced up-regulation of endothelial IL-8 remains unclear. In this study, PIECs treated with different agents were co-cultured with monocyte-derived dendritic cells (MoDCs), and the resulting DCs were analyzed using gene chip, western blot, confocal microscopy, and quantitative PCR methods. Specific signaling pathway inhibitors were used to validate the findings. This study aimed to investigate the signaling pathways associated with DC maturation inhibition mediated by PCV2-induced endothelial IL-8. Results showed that the NF-κB signaling pathway was the main pathway involved in DC maturation inhibition, which has provided improved scientific understanding of PCV2 infection-induced suppression of immune function.

## 2. Materials and Methods

### 2.1. Virus, Cells and Animal

The PCV2 strain SD/2008 (GenBank accession number GQ174519) was originally isolated from a piglet with clinical PMWS by the Laboratory of Animal Infectious Diseases of Hebei Agricultural University.

PIECs were purchased from the Cell Resource Center of Shanghai Institutes for Biological Sciences (Shanghai, China, Catalog number: GN105). The cells were cultured in 24-well plates in RPMI-1640 (GIBICO, Grand Island, NY, USA) complete medium (containing 10% heat-inactivated fetal bovine serum (FBS) (Sigma, St. Louis, MO, USA) and 200 U/mL of penicillin and 200 μg/mL of streptomycin). PIECs over-expressing IL-8 (IL-8^over^-PIECs) were constructed by our laboratory and cultured in 24-well plates using the same culture medium as the PIECs.

Six healthy 4-week-old White-Dutch Landrace crossbred piglets were obtained from the Beijing Centre of SPF Swine Breeding and Management. They were free of PCV2, PCV1, porcine reproductive and respiratory syndrome virus (PRRSV), porcine parvovirus (PPV), and classical swine fever virus (CSFV) per PCR/RT-PCR assessment, and were seronegative for antibodies against PCV2.

### 2.2. Purification of CD14^+^ Monocytes and Induction of MoDCs

Peripheral blood mononuclear cells (PBMCs) were isolated from healthy piglets using Ficollpaque (1.077 g/mL; TBD, Tianjin, China). CD14^+^ monocytes were then selected using CD14 monoclonal antibody labeled with FITC (Bio-Rad, Hercules, CA, USA) and isolated using magnetic beads (Miltenyi, Bergisch-Gladbach, Germany).

The purified CD14^+^ monocytes were resuspended in MoDC induction medium (complete medium containing 5 ng/mL porcine granulocyte-macrophage colony-stimulating factor (GM-CSF) (R&D, Minneapolis, MN, USA)) and 20 ng/mL recombinant porcine IL-4 (ProSpec, Rehovot, Israel), and half of the medium was changed every other day for 5 days. The cells were cultured in a humidified 5% CO_2_ atmosphere at 37 °C.

### 2.3. PCV2-Infected PIECs (PCV2-PIECs)

The PIECs in the logarithmic growth phase were seeded (1 × 10^5^ cells/well) into a 24-well culture plate. When PIECs reached 80% confluency, they were infected with PCV2 at a multiplicity of infection (MOI) of 0.5 in a humidified 5% CO_2_ atmosphere at 37 °C for 1 h. The cells without PCV2 in complete medium were used as a negative control. All of the experiments were performed in triplicate. The supernatant was then removed, and the cells were washed three times with PBS and cultured in 500 μL of complete medium under the same conditions as mentioned above for 24 h.

### 2.4. IL-8 Gene-Silenced PIECs (IL-8^si^-PIECs)

Briefly, 2 μL of Lipofectamine 2000 (Invitrogen, Carlsbad, CA, USA) and 80 μM of IL-8^si^ (Gene Pharma, Shanghai, China) were added into 24-well transwell plates with pre-plated PIECs in a serum-free RPMI 1640 medium. After 6 h, the serum-free medium was replaced by complete medium and incubated for 24 h. The transfection ratio was detected using fluorescence microscopy, where 60% fluorescence indicated successful transfection. IL-8 small interfering RNA (siRNA, sense: 5′-CGAUGCCAGUGCAUAAAUATT-3′, antisense: 5′-UAUUUAUGCACUGGCAUCGTT-3′) and negative (control) siRNA (sense: 5′-UUCUUCGAACGUGUCACGUTT-3′, antisense: 5′-ACGUGACACGUUCGGAGAATT-3′) were designed and synthesized by Shanghai Genepharma (Shanghai, China).

### 2.5. Co-Culture System

Co-cultures of cells were performed using transwell cell culture plates (Corning, Corning, NY, USA) with transwell membranes (0.1-μm pore size) (Millipore, Burlington, MA, USA). PIECs alone, PCV2-PIECs, IL-8^si^-PIECs, and IL-8^over^-PIECs were cultured in the lower chambers of the transwell plate individually at 37 °C for 24 h. MoDCs induced by the induction medium were seeded into the upper chamber of the transwell membranes. The cells were co-cultured in a humidified 5% CO_2_ incubator at 37 °C for 48 h. The ratio of cells in the upper chamber and lower chamber was 1:10. MoDCs co-cultured with different PIECs (DCs, PIECs-DCs, PCV2-PIECs-DCs, IL-8^si^-PIECs-DCs, and IL-8^over^-PIECs-DCs) were collected for further study.

### 2.6. Gene Chip Detection

Differently cultured DCs were sent to Shanghai Biotechnology company for Agilent Porcine Whole Genome 4*44K chip (design ID:026440) detection. Pathway analysis of the chip results was performed using the DAVID online analysis website. The *p* value < 0.05 was chosen as the critical value to select the significant KEGG signaling pathway related to the maturation of DCs. According to pathway and GO analysis results, the signaling pathways related to DC maturation in different treatment groups were screened.

### 2.7. Western Blot

Differently cultured DCs were lysed in RIPA lysate with protease inhibitor for 30 min on ice. Then, the proteins were separated using sodium dodecyl sulfate-polyacrylamide gel electrophoresis (SDS-PAGE) and transferred to polyvinylidene difluoride (PVDF) membranes (Beyotime Institute of Biotechnology, Beijing, China). After blocking with 5% nonfat milk at room temperature for 2 h, the membranes were hybridized with specific antibodies overnight at 4 °C. The membranes were then washed three times with TBST buffer and incubated with secondary antibodies at room temperature for 1 h. The results were visualized using a C-Digit chemiluminescence scanner (LI-COR, Lincoln, NE, USA). The antibodies used were as follows: NF-κB p65 (L8F6) mouse mAb (CST, Danvers, MA, USA), IκBα (L35A5) mouse mAb (CST, Danvers, MA, USA), beta Actin mouse mAb (Proteintech, Rosemont, IL, USA), HRP conjugated goat anti-mouse IgG (Proteintech, Rosemont, IL, USA), rabbit anti-porcine IκBα antibody (CST, Danvers, MA, USA), rabbit anti-porcine NF-κB p65 antibody (CST, Danvers, MA, USA), mouse anti-rabbit IgG-Alexa FluorR 647 (CST, Danvers, MA, USA), rabbit anti-porcine JAK2 antibody (Immunoway, Plano, TX, USA), rabbit anti-porcine p-JAK2 antibody (Immunoway, Plano, TX, USA), and rabbit anti-porcine STAT3 antibody (Immunoway, Plano, TX, USA).

### 2.8. Confocal Microscopy

Differently cultured DCs were fixed with 2% paraformaldehyde at room temperature for 15 min, washed three times with PBS, and then blocked with 5% bovine serum in PBS containing 0.1% Triton X-100 (Solarbio, Beijing, China) at room temperature for 1 h. Then, these cells were incubated with the NF-κB p65 (L8F6) mouse mAb (CST, Danvers, MA, USA) or rabbit anti-porcine p-STAT3 antibody (CST, Danvers, MA, USA) at 4 °C overnight. After washing three times with PBS, these cells were further incubated with anti-mouse IgG (Alexa Fluor^®^ 647 Conjugate) secondary antibody (CST, Danvers, MA, USA) or sheep anti-rabbit IgG-Alexa Fluor 555 (Invitrogen, Carlsbad, CA, USA) at room temperature for 1 h in the dark. Finally, nuclear cells were labeled with DAPI (Solarbio, Beijing, China) at 37 °C for 20 min and washed three times with PBS. Nuclear transfer of NF-κB p65 was observed and imaged using an Olympus FV1000S laser confocal microscope (Olympus, Tokyo, Japan).

### 2.9. Signal Pathway Inhibitor Treatment

To screen the optimal concentration of inhibitors, the induced DCs were cultured in complete medium with 2 μM, 5 μM, 10 μM, or 20 μM of BAY 11-7082 (Beyotime, Shanghai, China) or Ruxolitinib (Selleck Chemicals, Houston, TX, USA). After 1 h of incubation, the culture medium was discarded, washed three times, and then 10% RPMI1640 induction medium was added to the upper chamber of the transwell membranes for a further 48 h. These MoDCs were collected to detect IL-12 mRNA and determine their optimal concentration. DCs treated at the optimal concentration were co-cultured with PIECs of different treatments. To minimize the impact of other factors, we established an IL-8 antibody panel by adding IL-8 antibody (Abcam, Cambridge, MA, USA) at a final concentration of 5 μg/mL to the co-culture. The six groups in the NF-κB signaling pathway experiment included PCV2-PIECs-DCs, IL-8Ab-PCV2-PIECs-DCs, IL-8Ab-PCV2-PIECs-BAY-DCs, IL-8^over^-PIECs-DCs, Ab-IL-8^over^-PIECs-DCs, and Ab-IL-8^over^-PIECs-BAY-DCs. The six groups in the JAK-STAT signaling pathway experiment included PCV2-PIECs-DCs, IL-8Ab-PCV2-PIECs-DCs, IL-8Ab-PCV2-PIECs-RUX-DCs, IL-8^over^-PIECs-DCs, Ab-IL-8^over^-PIECs-DCs, and Ab-IL-8^over^-PIECs-RUX-DCs. The DCs of different groups were used for flow cytometry and quantitative real-time PCR.

### 2.10. Flow Cytometry

Each group of MoDCs was suspended with 100 μL PBS on an individual bases. One μL of mouse anti-porcine FITC-SLA-DR antibody (AbD Serotec, Kidlington, UK) and R-PE-CD152(CTLA-4)-muIg (Ancell, Chatsworth, CA, USA) were added to each group of MoDCs and incubated in the dark on ice for 30 min. Then, 1 mL PBS was added to each cell and centrifuged at 1500 r/min for 5 min. Precipitated cells resuspended with 300 μL PBS were prepared on an Attune NxT flow cytometer (Invitrogen, Carlsbad, CA, USA) and analyzed using FlowJo software (Tree Star Inc., Ashland, OR, USA). SLA Class II DR-FITC and CD152 double positivity represented the maturation of MoDCs.

### 2.11. Quantitative Real-Time PCR (qPCR)

Cell samples were harvested, and total RNA was extracted with Trizol reagent (Invitrogen, Carlsbad, CA, USA). Reverse transcription was carried out according to the instructions of the applied reverse transcription kit (Cwbio, Beijing, China). The reverse transcription product was used as a template. IL-12 specific primers (sense: 5′-ATGCTGGCCAGTACACC-3′, anti-sense: 5′-TCCAGCACGACCTCAATG-3′) and GM-CSF specific primers (sense: 5′-AGCCCTGAGCCTTCTAAAC-3′, anti-sense: 5′-CAAAGGGGATGGTAAAAAGA-3′) were used to detect their gene expression. The relative expression levels were normalized to the beta-actin level using the 2^−ΔΔCT^ method. qPCR reaction conditions were as follows: 95 °C for 10 min, followed by 40 cycles of 95 °C for 30 s, 59 °C (IL-12) or 62 °C (GM-CSF) for 30 s, and 72 °C for 30 s.

### 2.12. Ethics Statement

The treatment of all animals was handled in accordance with the ethical approval of Beijing University of Agriculture (Approval Number SYXK (BUA) 2021-0006). The protocol was approved by the Beijing Administration Office of Laboratory Animal Care and Ethics Committee.

### 2.13. Statistical Analysis

All statistical analysis was performed using SPSS Statistics 17 (IBM Corporation, Armonk, NY, USA). The results were expressed as the mean ± standard deviation (SD). Differences between groups were analyzed using ANOVA, followed by Duncan’s multiple range test for multiple comparisons. *p* values < 0.05 were considered to indicate significant differences, and those <0.01 were considered to indicate very significant differences. Unless indicated otherwise, the experiments were performed in triplicate (n = 3).

## 3. Results

### 3.1. Chip Analysis of Signaling Pathway of MoDCs Maturation Affected by PCV2-Induced Endothelial IL-8

MoDCs obtained from co-culture of different PIECs were subjected to microarray detection. GO and pathway analyses were performed for gene expressions with more than two-fold differences. By comparing between PIECs-DCs and DCs, PCV2-PIECs-DCs and PIECs-DCs, IL-8^si^-PIECs-DCs and PIECs-DCs, and IL-8^over^-PIECs-DCs and PIECs-DCs, respectively, genes with relative expression rates exceeding two (*p* < 0.05) were identified through chip analysis (Supplementary Table S1). The analysis revealed that 3448 genes exhibited a more than two-fold change in relative expression ratio (*p* < 0.05) between PIECs-DCs and DCs, with 1815 genes up-regulated and 1632 genes down-regulated. Similarly, between PCV2-PIECs-DCs and PIECs-DCs, 3105 genes showed a more than two-fold change in relative expression ratio (*p* < 0.05), with 1482 genes up-regulated and 1622 genes down-regulated. For IL-8^si^-PIECs-DCs and PIECs-DCs, 2329 genes displayed a more than two-fold change (*p* < 0.05), with 1375 genes up-regulated and 954 genes down-regulated. Moreover, for IL-8^over^-PIECs-DCs versus PIECs-DCs, 3413 genes showed a more than two-fold change (*p* < 0.05), with 1791 up-regulated genes and 1622 down-regulated genes. Then, GO analysis was performed on these genes (Supplementary Table S2). The results showed that differential genes were mainly involved in functions such as immune response, antigen presentation, inflammatory response, angiogenesis, and cell adhesion. The comparison of pathway results is shown in Table 1. Compared to the PIECs-DCs group, the factors of the NF-κB signaling pathway in the IL-8^over^-PIECs-DCs and PCV2-PIECs-DCs groups, such as the key molecules TAK1-binding protein (TAB), IKKα, RELA, and their downstream genes IL-8 and IL1β, were significantly down-regulated, whereas IκBα expression was significantly increased. However, in the IL-8^si^-PIECs-DCs group, the expression of the key molecules of NF-κB signaling pathway, such as TAB, IKKα, RELA, and their downstream genes (IL1β, etc.), were significantly up-regulated, and IκBα was significantly down-regulated. These results suggested that the NF-κB signaling pathway may be involved in the signaling mechanism of MoDC maturation inhibition by PCV2-induced endothelial IL-8. At the same time, the factors of the JAK2-STAT3 signaling pathway, such as JAK2, STAT2, STAT3, and IL-12A, were significantly down-regulated in the PIECs-DCs group compared to the DCs group (Table 2). Compared with the PIECs-DCs group, IL23A, IFNB1, JAK2, STAT3, STAT5, IL12A, IL12B, and IL7 in the PCV2-PIECs-DCs group showed significant down-regulation. IL2B, JAK, STAT2, STAT5, IFNGR1, IL23A, IL12RB1, and IL7 in the IL-8^over^-PIECs-DCs group were significantly lower, while in the IL-8^si^-PIECs-DCs group, the AKT3, IL2RA, JAK, STAT2, STAT5, IL23A, IL12RB1, and IL7 factors were significantly up-regulated. All of the above indicated that the JAK2-STAT3 signaling pathway may also be involved in MoDC maturation inhibition by PCV2-induced endothelial IL-8.

### 3.2. The NF-κB Signaling Pathway Was the Main Signaling Pathway for MoDC Maturation Inhibited by PCV2-Induced Endothelia IL-8

**Figure 1:**
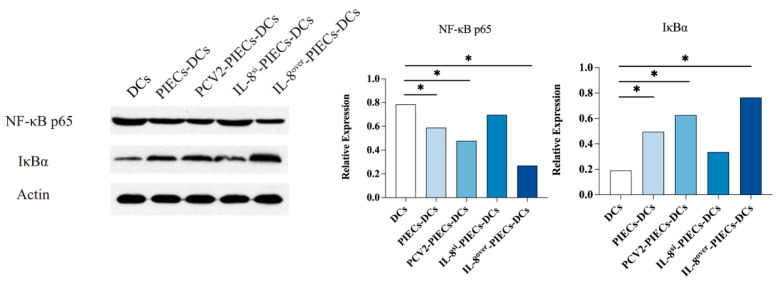
PCV2-induced endothelial-derived IL-8 inhibited IκBα and NF-κB p65 activity in MoDCs. Total protein of MoDCs was extracted and resolved using SDS-PAGE and transferred to PVDF membranes, which were hybridized with specific antibody and secondary antibody. The expression of NF-κB p65 and IκBα were analyzed. Error bars represent the standard deviation. * *p* < 0.05.

Based on the molecular changes in the signaling pathway of DC maturation at the gene level, western blot analysis was performed to detect the expression of NF-κB p65 and IκBα in MoDCs at the protein level (Figure 1). Compared with PIECs-DCs, the expression of NF-κB p65 was significantly decreased, and the expression of IκBα was significantly increased, in IL-8^over^-PIECs-DCs and PCV2-PIECs-DCs. Meanwhile, the expression of NF-κB p65 was significantly increased and the expression of IκBα was significantly decreased in IL-8^si^-PIECs-DCs. All of these results indicated that PCV2-induced endothelial IL-8 inhibited the degradation of IκBα and the activation of NF-κB p65 in MoDCs, which further suppressed the maturation of MoDCs.

**Figure 2:**
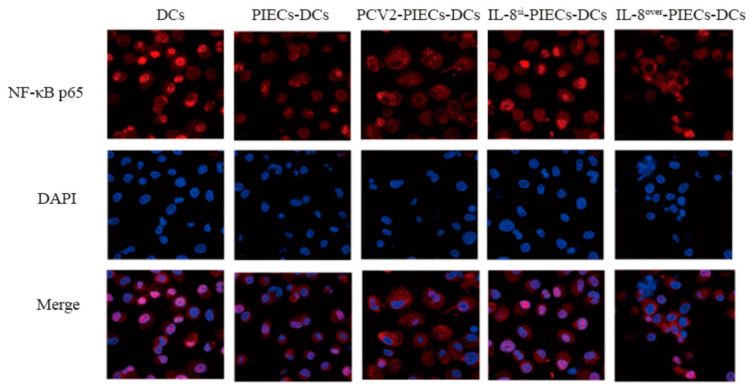
PCV2-induced endothelial-derived IL-8 inhibited the nuclear transfer of NF-κB p65 in MoDCs. Nuclear translocation of NF-κB p65 was observed using laser confocal microscopy across different groups. The localization of NF-κB p65 (red) was observed by a microscope using immunofluorescence stain with NF-κB p65 (L8F6) mouse mAb antibody and anti-mouse IgG secondary antibody. Nuclei were visualized via staining with DAPI (4′,6′-diamidino-2-phenylindole).

Considering that cells were stimulated by external factors, intracellular IκBα was activated and further degraded, while NF-κB p65, inhibited by IκBα, was simultaneously activated and underwent nuclear translocation to induce transcription of target genes [17]. Therefore, in this study, NF-κB p65 nuclear translocation of MoDCs co-cultured with different PIECs was analyzed using laser confocal microscopy (Figure 2). The results showed obvious NF-κB p65 aggregation in the nucleus of the PIECs-DCs compared to that in the DCs, while NF-κB p65 was widely dispersed in the cytoplasm in the PCV2-PIECs-DCs and the IL-8^over^-PIECs-DCs, respectively, compared with that in PIECs-DCs. These results suggested that PCV2-induced endothelial IL-8 could inhibit nuclear translocation of NF-κB p65 in MoDCs.

### 3.3. PCV2-Induced Endothelial IL-8 Inhibited the Expression of IL-12 and GM-CSF in MoDCs by NF-κB Signaling Pathway

**Figure 3:**
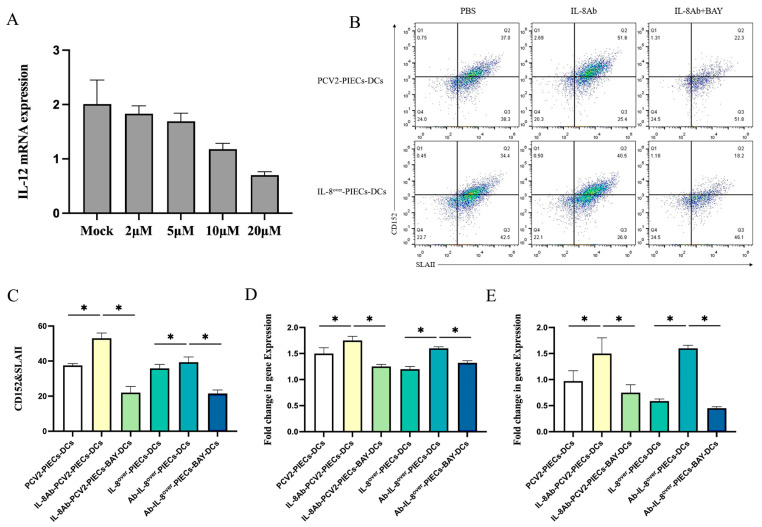
Inhibition of NF-κB signaling pathway weakened the maturity of MoDCs, and PCV2-induced endothelial-derived IL-8 inhibited the expression of IL-12 and GM-CSF in MoDCs via the NF-κB signaling pathway. (**A**) Induced MoDCs were pretreated with of a final concentration of 2 μM, 5 μM, 10 μM, or 20 μM of BAY 11-7082, and then co-cultured with different IPECs for 48 h. Relative IL-12 mRNA expression was detected using qPCR. (**B**,**C**) SLAII^+^CD152^+^ cells of MoDCs (BAY treated or not) 2 days after co-culture with different PIECs were analyzed using flow cytometry. IL-8 overexpressed PIECs were used as the positive control. Representative data from multiple experiments are shown in the scatter plot. (**D**) The expression of IL-12 at mRNA level in different groups, detected by real-time qPCR. (**E**) The expression of GM-CSF at mRNA level in different groups, detected by real-time qPCR. The data are results of three independent experiments of relative expression values compared with β-actin mRNA, and are represented as the mean and standard deviation (error bars) for each group. * *p* < 0.05 compared to the control.

IL-12, as a Th1 type cytokine, is highly expressed in mature DCs, and GM-CSF is an important cytokine induced by DCs [18]. Considering the importance of these two cytokines in the function of mature DCs, the expression of IL-12 in DCs at mRNA level was detected using qPCR. Firstly, to select the optimal concentration of the NF-κB signaling pathway inhibitor, MoDCs were taken after 5 days of induction culture and were pretreated with various concentrations of BAY 11-7082, and then co-cultured with different IPECs for 48 h. The mRNA level of IL-12 decreased in a dose-dependent manner (Figure 3A). It was found that 10 μM of BAY 11-7082 could significantly inhibit the activation of the NF-κB signaling pathway, which in turn inhibited the expression of downstream genes; thus, 10 μM of BAY 11-7082 was selected for further experiments.

Double positivity of CD80/86 and MHC II represent the maturity of antigen presenting cells [19]; therefore, in the present experiment, percentage amounts of SLA Class II DR and CD152 double positive cells were detected using flow cytometry. The results showed that the maturity of MoDCs in the IL-8Ab-PCV2-PIECs-DCs group was significantly higher than that in the IL-8Ab-PCV2-PIECs-BAY-DCs group (*p* < 0.05) (Figure 3B,C). The above results indicated that inhibition of the NF-κB pathway could weaken the maturation of MoDCs.

MoDCs, after 5 days of induction culture, were pretreated with 10μM of BAY 11-7082 and then co-cultured with different IPECs for 48 h. MoDCs co-cultured with different PIECs were collected, and qPCR detection results (Figure 3D,E) showed that the expression of IL-12 and GM-CSF at mRNA levels in IL-8Ab-PCV2-PIECs-DCs and Ab-IL-8^over^-PIECs-DCs was significantly higher than those in PCV2-PIECs-DCs and IL-8^over^-PIECs-DCs. Additionally, the mRNA expression of IL-12 and GM-CSF in IL-8Ab-PCV2-PIECs-BAY-DCs and Ab-IL-8^over^-PIECs-BAY-DCs was significantly lower than those in IL-8Ab-PCV2-PIECs-DCs and Ab-IL-8^over^-PIECs-DCs. The lower expressions of IL-12 and GM-CSF mRNA in MoDCs might be attributed to the maturation of MoDCs inhibited by PCV2-induced endothelial IL-8 via the NF-κB signaling pathway.

### 3.4. JAK2-STAT3 Signaling Pathway Did Not Significantly Affect the Maturation of MoDCs, Although the Up-Regulated Endothelial IL-8 by PCV2 Induced Nuclear Translocation of p-STAT3 in MoDCs

**Figure 4:**
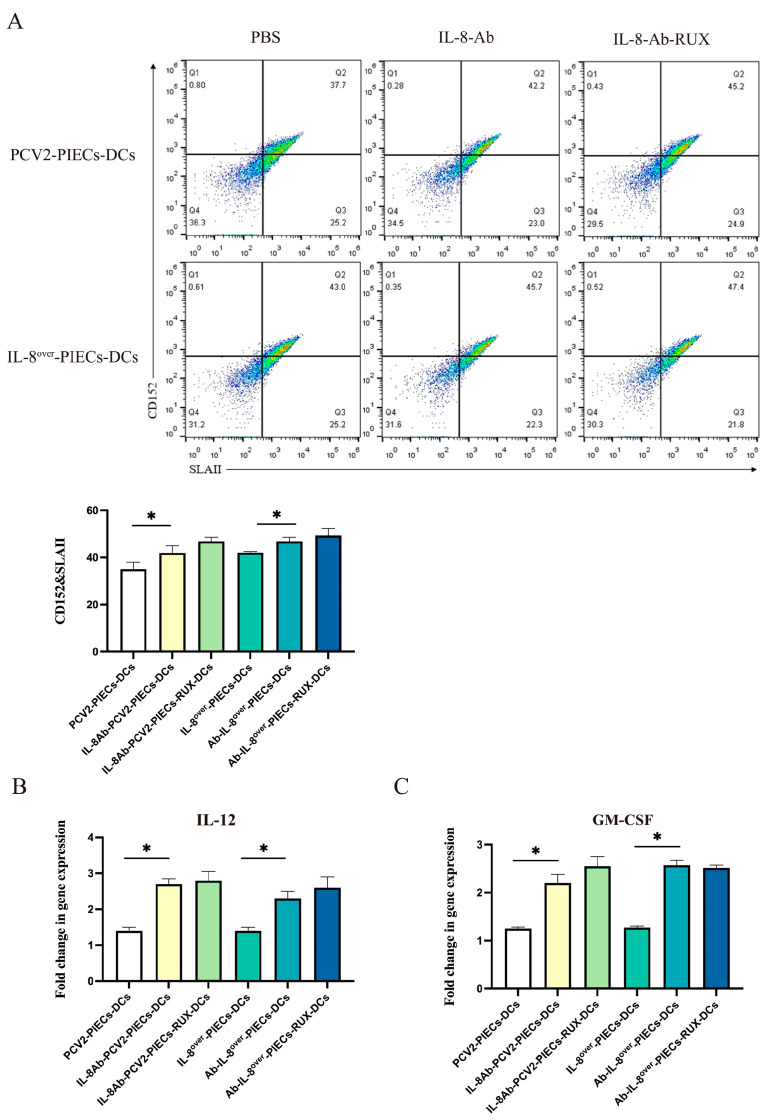
JAK2-STAT3 signaling pathway had no significant effect on the maturation of MoDCs. (**A**) Percentage of SLAII^+^CD152^+^ cells of MoDCs (RUX treated or not) 2 days after co-culture with different PIECs, analyzed using flow cytometry. IL-8 over-expressing PIECs were used as the positive control. Representative data from multiple experiments are shown in the scatter plot. (**B**,**C**) Expressions of IL-12 and GM-CSF of MoDCs 2 days after co-culture with different PIECs, detected by qPCR. The data are results of three independent experiments of relative expression values compared with β-actin mRNA, and are represented as the mean and standard deviation (error bars) for each group. * *p* < 0.05 compared to the control.

**Figure 5:**
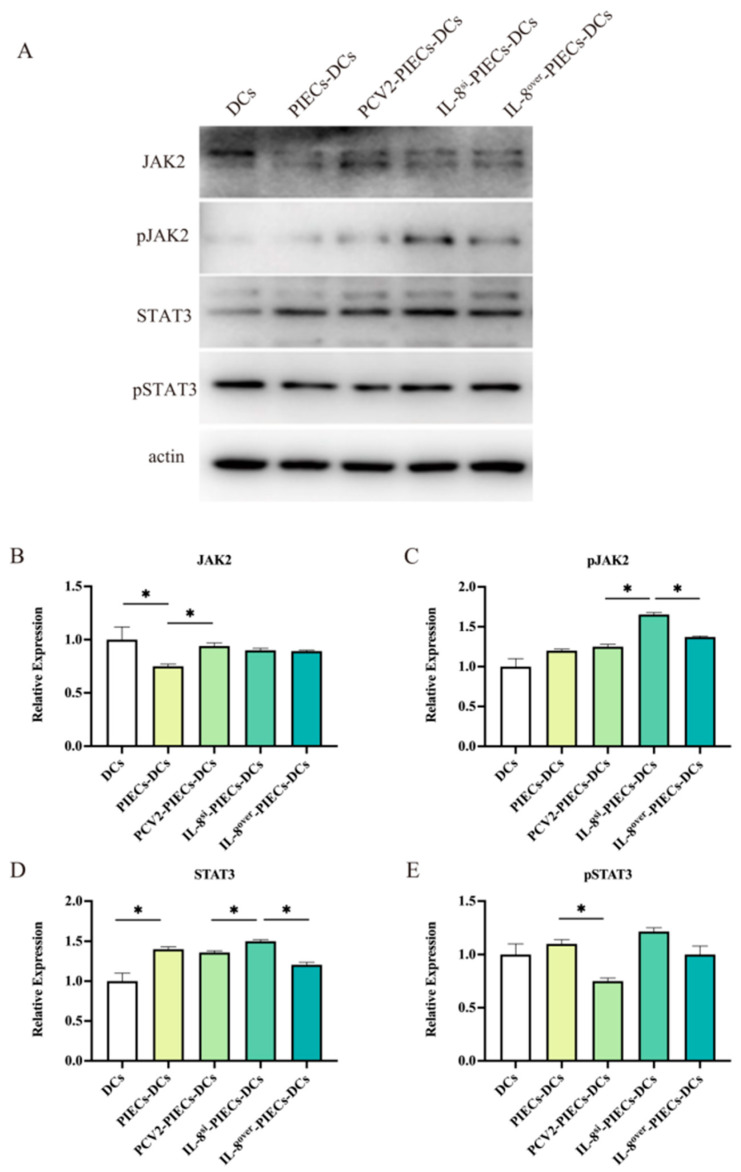
The expression of JAK2, pJAK2, STAT3, and pSTAT3 at the protein level of MoDCs, induced by PCV2 infected PIECs at the protein level. (**A**–**E**) MoDCs were collected after 2 days of co-culture with different PIECs and analyzed using native PAGE, followed by western blotting with anti-JAK2, pJAK2, STAT3, and pSTAT3 mAb. The expressions of JAK2 (**B**), pJAK2 (**C**), STAT3 (**D**), and pSTAT3 (**E**) were analyzed. Data are results of three independent experiments and are presented as the mean and standard deviation (error bars) for each group. * *p* < 0.05 compared to the control.

**Figure 6:**
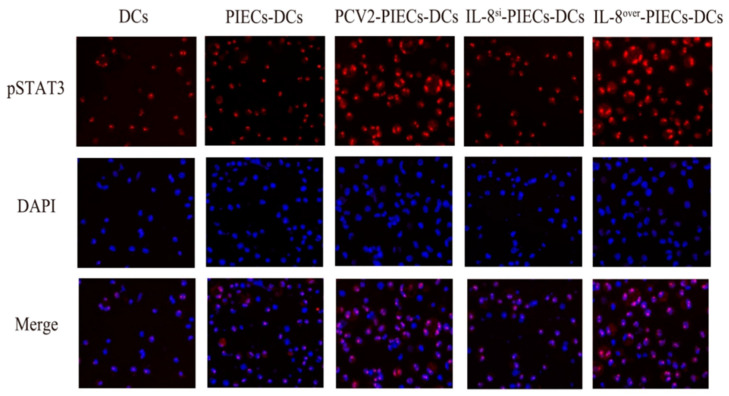
Nuclear translocation of pSTAT3 of MoDCs 2 days after co-culture with different PIECs. Cellular localization of pSTAT3 in MoDCs was measured using a confocal microscope. The localization of pSTAT3 (red) was observed via a microscope using immunofluorescence stain with anti-pSTAT3 and Alexa Fluor555 conjugate anti-rabbit IgG. Nuclei were stained with DAPI. Bar = 20 μm.

According to the microarray results, the JAK2-STAT3 signaling pathway in MoDCs might also be regulated by endothelial IL-8 induced by PCV2-infected PIECs. To demonstrate the role of the JAK2-STAT3 signaling pathway in MoDC maturation, MoDCs treated with Ruxolitinib were co-cultured with different PIECs. Flow cytometry analysis showed (Figure 4A) that there was no significant difference in MoDC maturation between the IL-8-PCV2-PIECs-RUX-DCs and the IL-8-PCV2-PIECs-DCs. qPCR results showed (Figure 4B,C) that the IL-8-PCV2-PIECs-RUX-DCs and the IL-8-PCV2-PIECs-DCs also had no significant difference in mRNA expression of IL-12 and GM-CSF, indicating that the JAK2-STAT3 pathway had no significant effect. Our further studies showed that the expression of JAK2, p-JAK2, STAT3, and p-STAT3 exhibited no significant difference between PCV2-PIECs-DCs and PCV2-IL-8^si^-PIECs-DCs (Figure 5). Only laser scanning confocal results showed that p-STAT3 nuclear translocation was significantly higher in PCV2-PIECs-DCs than in PCV2-IL-8^si^-PIECs-DCs (*p* < 0.05) (Figure 6). These results suggested that endothelial IL-8 induced by PCV2 could only induce p-STAT3 nuclear translocation in MoDCs to a certain extent, and that the JAK2-STAT3 pathway is not the main pathway via which PCV2-induced endothelial IL-8 affects the maturation of MoDCs.

## 4. Discussion

The prevention of infectious diseases is crucial for reducing economic losses. PCV2 is considered one of the most important pathogens in pig populations worldwide [20]. The internalization of PCV2 in immune cells contributes to the spread of the virus in pigs and may regulate important immune cells, such as dendritic cells [21]. Previous studies in our laboratory have shown that PCV2-induced endothelial IL-8 could affect MoDC maturation and antigen-presenting function [12]. Based on this study, we further investigated the effects of PCV2-induced endothelial IL-8 on related signaling pathways of dendritic cell maturation.

The NF-κB family of transcription factors consists of RelA (p65), RelB, c-Rel, p50, and p52 [22]. The homodimers or heterodimer of these proteins binds to specific DNA sequences in the target gene promoter region, causing them to dimerize, thereby activating or inhibiting their transcription [23]. The transcription factor NF-κB is initially inactive in the cytoplasm due to the inhibitor protein IκB [24]. Encoded by NFKBIA, IκBɑ serves as the primary negative regulator of NF-κB activity, preventing NF-κB subunits from binding to DNA by keeping them out of the nucleus. The classical NF-κB pathway can be activated by various stimuli, including TNF-α, LPS, and IL-1β. Upon activation of the IκK complex, phosphorylation of IκBα occurs, leading to its degradation through the proteasome. This process ultimately results in the proteasomal degradation of IκBα and the subsequent release and translocation of NF-κB dimers, such as p50-RelA (p65), into the nucleus to initiate the transcription of target genes [25]. Knockdown of the NF-κB activating protein IκKβ has been shown to significantly inhibit dendritic cell maturation and migration [26,27]. In the present study, firstly, the expression profiles of MoDC genes in different co-culture groups were compared. According to GO analysis, different genes were mainly involved in the functions of immune response, antigen presentation, inflammatory response, angiogenesis and cell adhesion, etc. Pathway analysis showed that the NF-κB related factor RELA decreased significantly in the IL-8 up-regulation group (PIECs-DCs to DCs, PCV2-PIECs-DCs to PIECs-DCs, IL-8^over^-PIECs-DCs to PIECs-DCs), while in the IL-8 down-regulation group (IL-8^si^-PIECs-DCs to PIECs-DCs), it increased significantly, which indicated that PCV2-induced endothelial IL-8 could affect the expression of RELA in MoDCs. Secondly, results relating to MoDCs co-cultured with different PIECs showed that the expression of NF-κB p65 decreased significantly and the expression of IκBα increased significantly at the protein level in the IL-8 up-regulated group, indicating that PCV2-induced endothelial IL-8 could suppress the NF-κB signaling pathway. Finally, aggregation of NF-κB p65 in the nucleus was clearly observed in the IL-8 down-regulated group, while in the IL-8 up-regulated group, NF-κB p65 was widely distributed in the cytoplasm, indicating that endothelial IL-8 induced by PCV2 inhibited the nuclear translocation of NF-κB p65 in MoDCs. Additionally, we also selected two key factors for MoDCs maturation: IL-12 and GM-CSF. The maturation of DCs treated with signaling pathway inhibitors was inhibited, and the expression of cytokines in each group of DCs was down-regulated. These results further proved that the inhibition of MoDC maturation by PCV2-induced endothelial IL-8 was influenced by the NF-κB signaling pathway.

When the cytokine bound to a receptor outside the cell membrane, the cytokine receptor was activated, resulting in transmission of the signal to JAK kinase, which was phosphorylated. The downstream molecule, STAT, was then further phosphorylated. Phosphorylated STAT can enter the nucleus and act as a part of the transcription factor complex, controlling the transcription of the cellular genes, thereby affecting the biological function of cells [28]. The immunosuppressant rapamycin greatly attenuates the immunostimulatory capacity of DCs by inhibiting IL-12-mediated activation of the JAK2/STAT4 signaling pathway in DCs [29]. STAT3 can regulate the development of tolerant DCs and inhibit the expression of the co-stimulatory molecules CD80 and CD86 [30]. Both IL-23R and IL-12R can activate JAK-STAT family members to mediate receptor-specific signaling [31]. When IFNAR1 binds to type I IFN, it can activate the JAK-STAT signaling pathway [32]. In the present study, the chip results showed that gene expressions with more than two-fold differences between MoDCs in different treatment groups were enriched in the JAK-STAT signaling pathway. The pathway analysis of differentially expressed genes between different MoDC treatment groups (PIECs-DCs to DCs, PCV2-PIECs-DCs to PIECs-DCs, IL-8^over^-PIECs-DCs to PIECs-DCs) showed that JAK2-STAT3 signaling pathway related factors (AKT3, IL2RA, JAK, STAT2, STAT5, IL23A, IL12RB1, and IL7) were down-regulated significantly, while those in the IL-8^si^-PIECs-DCs group were up-regulated significantly. Subsequently, western blotting showed that up-regulation of endothelial IL-8 by PCV2 could induce p-STAT3 nuclear translocation in MoDCs. However, after treatment with pathway inhibitors, the maturation of MoDCs was not inhibited, and the expression levels of IL-12 and GM-CSF mRNA did not change significantly, indicating that the JAK2-STTAT3 pathway has little impact on the maturation of MoDCs.

## 5. Conclusions

In summary, the NF-κB signaling pathway is the main signaling pathway of dendritic cell maturation inhibited by PCV2-induced endothelial IL-8. This study elucidates the molecular mechanism of PCV2 immunosuppression from the perspective of the interaction between PCV2 infected endothelial cells and MoDCs.

## Figures and Tables

**Table 1:** Differential genes associated with NF-κB signaling pathway in different groups.

Group	Gene
PIECs-DCs vs. DCs	↑: ATM. BLNK. LOC396781. LCK. NFKBIA. LOC100626904. PLAU ↓: BCL2L1. CFLAR. DDX58. RELA. TNFRSF1A. CCL4. CHUK. LTB. PTGS2. TLR4. TNFSF11. TNF. VCAM1
PCV2-PIECs-DCs vs. PIECs-DCs	↑: CXCL12. LOC396781. TAB1. TRAF3. LOC100626904. IL1B1. IL1B2 ↓:CD40LG. NFKBIA. RELA. TRAF6. ICAM1. LOC100626904. LBP TLR4. TNFSF11
IL-8^over^-PIECs-DCs vs. PIECs-DCs	↑: CXCL12. BLNK. CXCL8. CD40LG. LOC396781. LCK.RELA. LOC100626904. LTA. TNFSF11. VCAM1. CHUK. ICAM1. IL1B1. MYD88. TAB1. TRAF3. TRAF6. CD40LG ↓: ATM. DDX58. CCL4. PLAU. PTGS2. TLR4. TNF. IRAK1. NFKBIA
IL-8^si^-PIECs-DCs vs. PIECs-DCs	↑: ATM. DDX58. CCL4. LBP. NFKB1. PLAU. PTGS2. TLR41. ↓: BLNK. BCL2. LOC396781. LCK. RELA. TNFRSF1A. IL1B2. LOC100626904. LTA. TNFSF11. TNF. VCAM1

Note: All genes *p* < 0.05 and fold change >2 compared to control group. PIEC, porcine iliac artery endothelial cells.

**Table 2:** Differential genes associated with JAK-STAT signaling pathway in different groups.

Group	Gene
PIECs-DCs vs. DCs	↑: SOCS3. PIM1. IL15. IL6R. LOC100511937. EPOR. IL7R. IL13RA2. IL13RA1. LIF. SOCS2. ↓:IL2RA. MYC. STAT2. BCL2L1. IL23A. IL19. JAK2. STAT3. IL12A. IL12B.
PCV2-PIECs-DCs vs. PIECs-DCs	↑:IL15. IL19. LIFR. PRLR. IL5RA. PRL ↓:IL23A. IFNB1. JAK2. IL12A. IL12B. IL7. SPRY2
IL-8^over^-PIECs-DCs vs. PIECs-DCs	↑:IL6R. 10RB. PRL. SOCS4. TYK2. IL13RA2. LIF. SOCS2. OSM ↓: AKT3. IL2RA. MYC. IL15. IL12B. JAK. STAT2. IFNGR1. IL23A. IL12RB1. IL7. IL19. LEP
IL-8^si^-PIECs-DCs vs. PIECs-DCs	↑: AKT3. IL2RA. IL15. JAK. IFNGR1. IL23A. IL10RB. PRL. STAT2. IL12B. LIF. IL12RB1. IL7. TYK2 ↓: MYC. IL6R. SOCS4. IL13RA2. SOCS2. IL19. LEP. OSM

Note: All genes *p* < 0.05 and fold change >2 compared to control group. PIEC, porcine iliac artery endothelial cells.

## Data Availability

Data are contained within the article and Supplementary Materials.

## Supplementary Materials

The following supporting information can be downloaded at: https://www.mdpi.com/article/10.3390/v16040646/s1. Table S1: Result of differentially expressed genes (*p* < 0.05); Table S2: GO analysis results of differentially expressed genes.
