# Supplementary material for: PCV2 Induced Endothelial Derived IL-8 Affects MoDCs Maturation Mainly via NF-κB Signaling Pathway
Source: Viruses. 2024 Apr 22;16(4):646. doi: 10.3390/v16040646 (PMC11053600; doi:10.3390/v16040646)
Supplement: Supplementary file 1 [file viruses-16-00646-s001.zip › viruses-2883734-supplementary.pdf]

Table S1. Result of differentially expressed genes ($P < 0.05$).

Group	Gene
**Immune response**	
PIECs-DCs vs DCs	↑: CCL24.CCR1. CXCL10. CXCL13. CXCL14. CD74. JUN. SLA-DQA1. LSA-DOB. XCL1. CCL14. CCL3L1. CCL4. CCR5. CXCL2. CSF2. IL-1A. IL-10. IL-6. TLR7.<br>↓: OAS1.OAS2. CCL17. CCL20.CCR7. CD36. CD70. LOC106504372. LOC100520439. SLA-3. SLA-DRA. SLA-DRB1. SLA-DMB. SLA-DMA. SLA-DQB1. CSF3.IL-12A.IL-12B.IL-15.IL-19. IL-2RA.IL-23A.THBS1. TNFSF10. TNFSF11. VTN.
PCV2-PIECs-DCs vs PIECs-DCs	↑: OAS1. CCL17. CCL20. CCL24. CXCL12. CXCL10. CXCL14. VTN. IL-6. IL-7. TGFBR3. IL-10.<br>↓: CCR2. CCR7. CCR4. CD36. CD70. CD74.SLA-DQA1. SLA-DRB1. SLA-DMB. SLA-DOB. SLA-DMA. SLA-DQB1. SLA-DRA. TNFSF11. IL-12B. CSF3. PPBP. THBS1. TNFSF10.
IL-8^{over}-PIECs-DCs vs PIECs-DCs	↑: CCL24. CXCL13. CXCL10. CXCL14. CD36. CD74. IL-6.<br>↓: OAS1. CCL20. CCR7. CD70. SLA-DQA1. SLA-DRB1. SLA-DMB. SLA-DOB. SLA-DMA. SLA-DQB1. AMCF-II. CSF3. IL-12B. IL-18. IL-2RA. IL-23A. IL-7. THBS1.
IL-8^{si}-PIECs-DCs vs PIECs-DCs	↑: CCL20. CCL5. CCR2. CXCL12. CD70.SLA-DQA1. SLA-DRB1. SLA-DMB. SLA-DMA. SLA-DQB1. SLA-DRA. XCL1. IL-12B. IL-12A.<br>↓: OAS2. CCL24. CXCL13. CXCL10. CXCL14. CD36. CD74. TNFAIP1. CCL14. IL-6. CCL8.
**Antigen processing and presentation of peptide or polysaccharide antigen via MHC class II**	
PIECs-DCs vs DCs	↑: LOC100520439.SLA-DOB.<br>↓: LOC106504372.SLA-DRA. SLA-DQA1.SLA-DRB1.SLA-DMB.SLA-DMA.SLA-DQB1.SLA-DOA.
PCV2-PIECs-DCs vs PIECs-DCs	↑: LOC100520439.<br>↓: LOC106504372. SLA-DRA. SLA-DQA1.SLA-DRB1.SLA-DMB.SLA-DMA.SLA-DQB1.SLA-DOA.SLA-DOB.
IL-8^{over}-PIECs-DCs vs PIECs-DCs	↑: LOC106504372<br>↓: LOC100520439. SLA-DQA1. SLA-DRB1. SLA-DMB. SLA-DMA. SLA-DQB1. SLA-DOB.
IL-8^{si}-PIECs-DCs vs PIECs-DCs	↑: LOC106504372. SLA-DRA. SLA-DQA1.SLA-DRB1.SLA-DMB.SLA-DMA.SLA-DQB1.<br>↓: LOC100520439.
**Inflammatory response**	
PIECs-DCs vs DCs	↑: CCR1. CXCL10.CXCL13. RELA. IL-10. TLR6. TLR7. TLR8.<br>↓: CCL17. CCL20. CCR7. CXCR6. JAK2. IL-17F. IL-19. IL-2RA. IL-23A. TLR2. TLR4.
PCV2-PIECs-DCs vs PIECs-DCs	↑: CCL17. CCL20. AMCF-II. CXCL10. CXCR6. TLR7.<br>↓: CCR2. CCR4. RELA. JAK2. IL-17B. IL-18. IL-23°. TLR8. TLR4.
IL-8^{over}-PIECs-DCs vs PIECs-DCs	↑: CXCL10. CCL4.<br>↓: CCL20. CCR2. CCR3. CCR7. RELA. TNFRSF1A.IL-23A. TLR1. TLR10. TLR8.
IL-8^{si}-PIECs-DCs vs PIECs-DCs	↑: CCL20. CCR2. CCL5. CCR3. CXCL12. RELA. IL-18. IL-23°. IL-2RA. TLR8.<br>↓: CXCL10. CXCL13. CCL4. CCL8. TLR3. TLR4.
**Cell adhesion**	

PIECs-DCs vs DCs	↑: CD2.<br>↓: THY1. ADGRG1. CERCAM. ITGA2.
PCV2-PIECs-DCs vs PIECs-DCs	↑: CD2. ITGB5.<br>↓: CASS4. THY1. WISP2. ADGRG1. ITGA2. ITGA6.
IL-8^{over}-PIECs-DCs vs PIECs-DCs	↑: CX3CR1. CASS4.<br>↓: CD2. THY1. ITGA6. THBS3.
IL-8^{si}-PIECs-DCs vs PIECs-DCs	↑: THY1. ADGRG1. ITGA6. ITGB5. ICAM1. ICAM3.<br>↓: CD2.
**Angiogenesis**	
PIECs-DCs vs DCs	↑:<br>↓: THY1. ADGRG1. ANG. ANGPT2. ANGPT4.
PCV2-PIECs-DCs vs PIECs-DCs	↑: PECAM1.<br>↓: TGFA. VEGFA. ANG. ANGPT2. THY1.
IL-8^{over}-PIECs-DCs vs PIECs-DCs	↑: PECAM1.<br>↓: THY1. ADGRG1. ANG. ANGPT4. VEGFA.
IL-8^{si}-PIECs-DCs vs PIECs-DCs	↑: THY1. ADGRG1. ANG. ANGPT4. VEGFA.<br>↓: ANGPT2.

Table S2. GO analysis results of differentially expressed genes.

Group	Item	Gene Number	Up-Regulated	Down-Regulated
**Biological process**				
PIECs-DCs vs DCs	cell-cell adherens junction	4	1	3
	positive regulation of angiogenesis cell adhesion	5	3	2
	cell adhesion	26	14	12
	Angiogenesis	10	6	4
	regulation of cell adhesion	19	11	8
	immune response	48	20	28
	I-kappaB kinase/NF-kappaB signaling	17	6	11
	adherens junction	12	5	7
	inflammatory response	24	11	13
	cell migration	24	8	16
	cell differentiation	63	34	29
	chemotaxis	11	6	5
PCV2-PIECs-DCs vs PIECs-DCs	immune response	30	14	16
	cell migration	17	8	9
	chemotaxis	11	4	7
	JAK-STAT cascade	9	4	5
	angiogenesis	11	3	8
	cell adhesion	17	5	12
IL-8^{over}-PIECs-DCs vs PIECs-DCs	immune response	46	17	29
	cell adhesion	22	8	14
	inflammatory response	25	7	18

	chemotaxis	14	4	10
	cell migration	21	6	17
	endocytosis	11	6	5
	JAK-STAT cascade	5	2	3
	cytokine-mediated signaling pathway	15	5	10
	I-kappaB kinase/NF-kappaB signaling	7	2	5
IL-8si-PIECs-DCs vs PIECs-DCs	immune response	49	27	22
	cell adhesion	21	15	6
	endocytosis	14	8	6
	cell migration	24	14	10
	axonogenesis	6	4	2
	I-kappaB kinase/NF-kappaB signaling	7	4	3
	chemotaxis	17	8	9
	JAK-STAT cascade	9	5	4
**Cellular component**				
PIECs-DCs vs DCs	cell-cell junction	10	6	4
	cell-cell adherens junction	4	3	1
	actin filament	4	3	1
	microtubule cytoskeleton	16	5	11
	actin cytoskeleton	13	6	7
PCV2-PIECs-DCs vs PIECs-DCs	actin cytoskeleton	11	8	3
	extracellular space	49	29	20
	cytoskeleton	23	10	13
	Adherens junction	8	3	5
IL-8over-PIECs-DCs vs PIECs-DCs	cell-cell adherens junction	4	2	2
	actin cytoskeleton	10	6	4
	cell junction	19	11	8
	cytoskeleton	31	21	10
IL-8si-PIECs-DCs vs PIECs-DCs	extracellular region	143	84	59
	cell-cell junction	7	4	3
	adherens junction	10	3	7
	cytoskeleton	25	10	15
	actin cytoskeleton	7	5	2
**molecular function**				
PIECs-DCs vs DCs	endopeptidase activity	17	6	11
	oxidoreductase activity, acting on a sulfur group of donors	5	3	2
	cytoskeletal protein binding	25	12	13
	transcription factor binding	9	3	6
	cytokine activity	19	8	11
PCV2-PIECs-DCs vs PIECs-DCs	transcription factor binding	6	2	4
	cytokine activity	13	4	9
	endopeptidase activity	11	3	8
	receptor activity	28	15	13
IL-8over-PIECs-DCs vs PIECs-DCs	cytokine binding	5	1	4
	transcription factor binding	5	2	3
	endopeptidase activity	21	13	7
	chemokine activity	4	3	1
IL-8si-PIECs-DCs	endopeptidase activity	19	12	7

vs PIECs-DCs	transcription factor binding	8	6	2
	oxidoreductase activity, acting on paired donors, with incorporation or reduction of molecular oxygen, NAD(P)H as one donor, and incorporation of one atom of oxygen	5	3	2
	cytokine activity	18	11	7
